# Minocycline-Mediated Inhibition of Microglial Activation in Neuroinflammation-Related Neuropsychiatric Disorders: Preclinical Promise and Clinical Limitations

**DOI:** 10.3390/ijms27198533

**Published:** 2026-09-24

**Authors:** Sadayuki Hashioka

**Affiliations:** Department of Psychiatry, Asahikawa Medical University, 2-1-1-1 Midorigaoka Higashi, Asahikawa 078-8510, Hokkaido, Japan; hashioka@f2.dion.ne.jp

**Keywords:** minocycline, microglia, neuroinflammation, neuropsychiatric disorders, Alzheimer disease, Lewy body disease, schizophrenia, major depressive disorder

## Abstract

Minocycline, a second-generation tetracycline antibiotic, has attracted interest as a CNS-active anti-inflammatory agent because it crosses the blood–brain barrier and inhibits microglial activation. This review summarizes preclinical and clinical evidence for minocycline in neuroinflammation-related neuropsychiatric disorders, focusing on Alzheimer’s disease (AD), Lewy body disease (LBD), schizophrenia, and major depressive disorder (MDD). Across these disorders, preclinical studies have generally shown that minocycline attenuates microglial activation or maladaptive microglial responses and improves disease-relevant behavioral, synaptic, or neuropathological outcomes. However, clinical translation has been inconsistent. Clinical trials in AD, related parkinsonian disorders, schizophrenia, and MDD have failed to demonstrate robust and reproducible efficacy, despite encouraging preclinical findings. Moreover, direct clinical evidence that minocycline suppresses microglial activation in the human brain remains very limited. These discrepancies between preclinical findings and clinical evidence may reflect heterogeneity in disease stage and microglial states, suboptimal timing of intervention, lack of direct evidence that minocycline suppresses microglial activation in the human brain, and its non-selective anti-inflammatory actions. Future studies should incorporate biomarker-based patient stratification and direct measures of central microglial target engagement.

## 1. Introduction

Minocycline is a second-generation semi-synthetic tetracycline antibiotic with broad-spectrum antibacterial activity. It is widely used as an antibiotic to treat skin infections, including acne vulgaris. Beyond its antimicrobial activity, minocycline exerts diverse biological effects, such as anti-inflammatory and anti-apoptotic actions (reviewed in [1,2]). These anti-inflammatory effects include inhibition of T-cell activation, prevention of neutrophil transmigration, and reduction in the release of proinflammatory cytokines and nitric oxide [2]. Accordingly, minocycline has occasionally been used off-label to treat sterile inflammatory conditions, such as rheumatoid arthritis [3].

Minocycline has also been considered as a CNS-active agent because of its ability to cross the blood–brain barrier, attributable to its high lipophilicity [4], and its capacity to inhibit microglial activation. In 1998, Koistinaho’s group first demonstrated that minocycline suppressed microglial, but not astrocytic, activation in a gerbil model of global ischemia [5]. Subsequently, in 2001, the same group demonstrated a direct inhibitory effect on pure microglial cultures: minocycline reduced excitotoxin-induced proliferation as well as microglial release of nitric oxide metabolites and interleukin (IL)-1β [6]. Since then, many studies have confirmed minocycline’s ability to inhibit microglial activation, although the precise molecular mechanisms remain unresolved. In addition, minocycline has been reported to exert anti-inflammatory effects on activated astrocytes [7]. Taken together, minocycline should be regarded not as a microglia-specific inhibitor, but as a non-selective inhibitor of microglial activation with broader anti-inflammatory actions.

Microglial activation is a well-documented pathological feature across a wide range of neuropsychiatric disorders. In the 1980s, postmortem brain studies showed that microglia in Alzheimer’s disease (AD) and Lewy body disease (LBD) lesions in the hippocampus and substantia nigra (SN), respectively, exhibited the amoeboid “macrophage-like” morphology indicative of an activated state [8,9]. In the 2010s, positron emission tomography (PET) studies using translocator protein (TSPO) radioligands provided in vivo evidence of altered neuroimmune signals not only in neurodegenerative disorders but also in psychiatric disorders, such as schizophrenia [10] and major depressive disorder (MDD) [11]. Although the details of how activated microglia contribute to the pathogenesis of the above neuropsychiatric disorders are still unclear, it is likely that chronic neuroinflammation is mediated, at least in part, by activated or maladaptive microglia.

Throughout this review, the term “microglial activation” is retained because it is widely used in the original literature reviewed here. However, it should not be interpreted as a single, uniform biological state. Depending on the study, microglial activation has been operationally defined by changes in morphology, microglial density or proliferation, expression of markers such as Iba-1, CD68, OX-42, OX-6, or TSPO, production of cytokines or nitric oxide, phagocytic activity, or inflammasome-related signaling. Contemporary transcriptomic and functional studies have shown that microglia exhibit diverse, context-dependent states that cannot be adequately captured by simple resting/activated or M1/M2 dichotomies [12,13]. Accordingly, when this review refers to minocycline as “inhibiting microglial activation,” the term denotes attenuation of inflammatory, maladaptive, or disease-relevant microglial responses as operationally defined in individual studies, rather than global suppression of microglial function. The putative mechanisms underlying the inhibitory effects of minocycline on microglial activation and their downstream consequences are summarized in Figure 1.

Based on the ability of minocycline to inhibit microglial activation associated with neuroinflammation, a growing number of studies have been conducted to evaluate its efficacy in various neuropsychiatric and neuroinflammatory disorders. Despite longstanding interest in minocycline as an anti-inflammatory modulator of microglial responses, relatively few original studies have been published in recent years, particularly studies demonstrating clear clinical translation. This relative paucity of recent evidence underscores the need to critically re-evaluate the available preclinical and clinical literature. This review summarizes the effects of minocycline on the two common neurodegenerative disorders that cause dementia, AD and LBD, as well as on two major psychiatric disorders, schizophrenia and MDD, with particular attention to both preclinical evidence and clinical translation.

## 2. Literature Search Strategy and Scope of the Review

This article is a narrative review rather than a systematic review or meta-analysis. Relevant literature was identified through iterative searches of PubMed, supplemented by manual screening of reference lists from key articles, clinical trials, and recent systematic reviews or meta-analyses. Searches were conducted using combinations of the following terms: “minocycline,” “microglia,” “microglial activation,” “neuroinflammation,” “Alzheimer’s disease,” “Lewy body disease,” “Parkinson’s disease,” “multiple system atrophy,” “schizophrenia,” “maternal immune activation,” “major depressive disorder,” “treatment-resistant depression,” “clinical trial,” “randomized controlled trial,” “PET,” “TSPO,” and “biomarker.” No lower publication-date limit was imposed. Searches were last updated in September 2026.

The review focused mainly on English-language preclinical studies, clinical trials, systematic reviews, and meta-analyses that examined minocycline in relation to microglial or neuroinflammatory mechanisms and disease-relevant or clinical outcomes. Since this was a narrative review, no formal prespecified inclusion or exclusion criteria were applied. Formal risk-of-bias assessment, quantitative evidence grading, and meta-analytic pooling were not performed.

AD, LBD and related parkinsonian disorders, schizophrenia, and MDD were selected because they represent major neurodegenerative and psychiatric conditions in which microglial or neuroinflammatory mechanisms have been implicated and in which both preclinical and clinical evidence for minocycline is available. The aim was to provide a critical synthesis of the available evidence rather than an exhaustive catalogue of all minocycline studies.

## 3. Effects of Minocycline on Alzheimer’s Disease

In vitro studies have demonstrated inhibitory effects of minocycline on amyloid β (Aβ)-activated microglia, suggesting a potential beneficial role in AD. Minocycline reduced secretion of IL-6, IL-1β and tumor necrosis factor (TNF)-α, excessive levels of which are potentially neurotoxic, from cultured human [14,15] and mouse [16] microglia stimulated with Aβ1-42, without preventing microglial phagocytosis of Aβ1-42. In addition, minocycline exerts direct neuroprotective, anti-amyloidogenic, and anti-tauogenic effects in vitro. For example, it reduced Aβ1-42-induced death of differentiated PC12 cells by inhibiting phosphorylation of eukaryotic initiation translation factor 2α [17], and it dose-dependently inhibited Aβ1-42 fibril formation in cultures of adult microglia from postmortem AD brains [14]. In primary rat cortical neurons, minocycline prevented Aβ1-42-induced neuronal death, blocked caspase-3 activation, and attenuated generation of tau fragments cleaved by caspase-3 [18].

Consistent with these in vitro findings, numerous in vivo studies using animal AD models support minocycline’s therapeutic potential. Most employed genetically modified mouse models, including mice transgenic for human amyloid precursor protein (APP) [16,19,20], APP/mutant human presenilin-1 (PS1)-double transgenic mice [21,22], and triple-transgenic mice expressing mutant APP, PS1, and tau [23]. Others used rats injected with human Aβ1-42 into the hippocampus [24,25] or ventricles [26,27]. Across the studies, minocycline consistently attenuated microgliosis and improved cognition and spatial learning and memory in behavioral assays such as the Morris water maze test, Barnes maze test, and novel object recognition tests. However, unlike in vitro findings, several in vivo studies reported no effect of minocycline on Aβ plaque burden [19,22] or tau hyperphosphorylation [23]. Importantly, severity of cognitive impairment has been shown to correlate more closely with synaptic dysfunction and glial activation than with Aβ burden [28]. Notably, Kater et al. (2023) demonstrated in *APP*/*PS1*-transgenic mice that oral minocycline treatment, when initiated before the onset of microgliosis, ameliorated synaptic deficits and cognitive decline, suggesting the importance of preventing microglial activation in the early phase [22]. These animal studies should be interpreted in light of important differences among experimental models. APP-transgenic, APP/PS1 double-transgenic, triple-transgenic, and Aβ-injection models capture overlapping but non-identical aspects of AD-related pathology, and they differ in the relative contribution of amyloid deposition, tau pathology, neuroinflammation, synaptic dysfunction, and neuronal injury. Among the in vivo studies reviewed here, most used amyloid-based models, whereas only one study employed a triple-transgenic model incorporating tau pathology. In addition, the age at which minocycline treatment is initiated, and whether treatment begins before or after the development of microgliosis, synaptic deficits, or cognitive impairment, may influence the observed outcomes. These factors may partly explain why minocycline improves cognition or synaptic measures in some models without consistently reducing Aβ plaque burden or tau hyperphosphorylation. Changes in Aβ levels or plaque burden should not necessarily be regarded as the sole criterion for therapeutic efficacy. Minocycline may influence inflammatory microglial responses, synaptic integrity, neuronal function, or behavioral outcomes without producing substantial changes in established amyloid burden. Conversely, reduction in amyloid pathology alone does not establish meaningful neuroprotection or clinical benefit.

Despite robust preclinical evidence, clinical data remain scarce. To date, only one randomized controlled trial (RCT) has evaluated minocycline in AD, without assessing its effects on microglial activation [29]. Disappointingly, in this two-year trial (n = 544), daily minocycline (200–400 mg) in patients with mild AD failed to produce meaningful benefits in cognition (Mini-Mental State Examination) or functional ability (Bristol Activities of Daily Living Scale).

The failure of minocycline in the AD clinical trial may reflect several translational limitations. First, animal models often involve genetically or experimentally induced pathology with relatively well-defined timing, whereas patients with mild AD already have heterogeneous and long-standing amyloid, tau, synaptic, vascular, and inflammatory changes. Second, minocycline may primarily modulate inflammatory microglial responses rather than directly modify established amyloid or tau pathology. Third, the trial did not directly assess whether minocycline suppressed disease-relevant microglial responses in the human brain. Therefore, the negative clinical result may reflect not only lack of clinical efficacy, but also limitations related to disease stage, patient selection, target engagement, and outcome assessment.

## 4. Effects of Minocycline on Lewy Body Disease

Evidence for minocycline in LBD and related parkinsonian disorders is limited and derives from heterogeneous experimental and clinical contexts. Preclinical studies have used toxin-induced PD models as well as α-synuclein-based models, whereas clinical evidence has mainly come from studies in PD and MSA. These models and disease entities are related but not interchangeable. MPTP, 6-OHDA, and intranigral LPS models are useful for examining dopaminergic neurodegeneration and inflammatory injury, whereas α-synuclein-based models more directly address Lewy body-related pathology. MSA, in contrast, is a distinct synucleinopathy characterized primarily by glial cytoplasmic inclusions and multisystem neurodegeneration. Accordingly, clinical findings in PD and MSA should be regarded as related parkinsonian evidence rather than as direct clinical evidence for LBD itself. While several in vivo studies have evaluated its effects in LBD-related models, only one in vitro study has been reported to date. In this study, minocycline inhibited 1-methyl-4-phenylpyridinium (MPP^+^)-induced microglial activation in BV2 cells, reducing caspase-1 and inducible nitric oxide synthase (iNOS) expression and protecting dopaminergic neurons in mixed neuron-glia cultures [30]. MPP^+^ is the toxic metabolite of MPTP and selectively accumulates in dopaminergic neurons via the dopamine transporter (DAT) [31].

Most LBD animal models rely on neurotoxin administration rather than genetic manipulation to induce sufficient nigrostriatal degeneration and motor symptoms. Neurotoxins include 1-methyl-4-phenyl-1,2,3,6-tetrahydropyridine (MPTP), which is converted to MPP^+^ and inhibits mitochondrial complex I, leading to ATP depletion and neuronal death [32,33], and 6-hydroxydopamine (6-OHDA), which is a selective substrate for DAT and induces oxidative stress-related cytotoxicity and mitochondrial impairment [34,35]. Lipopolysaccharide (LPS) injection into the SN has also been used, exploiting this region’s vulnerability to inflammatory injury [36]. LPS injection into the SN induced robust microglial activation that preceded the death of dopaminergic neurons [36]. Among these models, minocycline inhibited microglial activation and reduced dopaminergic neurodegeneration. For instance, in MPTP-treated mice, minocycline reduced mRNA levels of the microglial marker MAC-1 and microglial expression of inflammatory mediators (IL-1β, caspase-1, iNOS, and NADPH oxidase) and protected SN dopaminergic neurons [30,37]. In the 6-OHDA model, it reduced CD11^+^ microglia and preserved tyrosine hydroxylase-positive neurons [38]. In the LPS model, minocycline suppressed OX-42 and OX-6 immunoreactivity and decreased IL-1α and TNF-α mRNA, protecting dopaminergic neurons [39]. A recent study using α-synuclein-transgenic mice also showed that minocycline inhibited microglial activation, reduced IL-1β release, improved motor function, and attenuated Lewy body pathology [40]. In these models, most studies have demonstrated protective effects of minocycline against microglial activation and dopaminergic neurodegeneration, whereas only a few studies have reported neutral or deleterious outcomes including worsened outcomes in MPTP-treated mice [41] and monkeys [42].

Clinical trials investigating minocycline have not demonstrated consistent efficacy or safety in the treatment of LBD, especially Parkinson’s disease (PD). The NINDS NET-PD phase II trial suggested possible benefit [43]. However, follow-up showed no significant differences from placebo, with tolerability concerns leading to discontinuation in 23% of participants [44]. Another trial in multiple-system atrophy (MSA) of the parkinsonian type failed to improve motor function and had high dropout rates (72%) due to adverse effects such as gastrointestinal intolerance, worsening parkinsonism, and skin hyperpigmentation [45]. In this study, although ^11^C-PK11195-PET showed reduced microglial activation in some minocycline-treated patients, clinical benefits were lacking. Overall, the clinical relevance of microglial inhibition by minocycline in LBD remains uncertain.

## 5. Effects of Minocycline on Schizophrenia

Increased synaptic pruning by microglia has been recognized as a key mechanism in the pathogenesis of schizophrenia, particularly during adolescence [46,46,47]. Two seminal in vitro studies have demonstrated inhibitory effects of minocycline on microglial synapse pruning and implied potential therapeutic efficacy of the drug in schizophrenia. Sellgren et al. (2019) [48] showed that induced microglia-like cells (iMGs) derived from patients with schizophrenia exhibited significantly increased phagocytosis of synaptosomes compared with control iMGs. Moreover, synaptosomes isolated from schizophrenia patient-derived induced pluripotent stem (iPSC) neurons, which show enhanced C3 deposition associated with higher C4A expression, were also more readily engulfed than those from controls [48]. Minocycline decreased the synapse engulfment at clinically relevant concentrations in both schizophrenia-derived and control iMGs [48]. Breitmeyer et al. (2023) [49] demonstrated in a co-culture system that schizophrenia patient-derived iPSC neurons, which intrinsically exhibit reduced presynaptic density, and patient-derived microglia in an inflammatory activation state synergistically interacted to exacerbate synaptic loss. This interaction resulted in a marked increase in the microglial engulfment of presynaptic structures [49]. Furthermore, minocycline pretreatment attenuated microglial activation and selectively rescued presynaptic density in schizophrenia-derived co-cultures, while exerting minimal effects in control co-cultures [49]. These in vitro studies strongly suggest that a pharmacological approach targeting microglia-mediated synaptic loss could represent a therapeutic strategy to delay the progression, or even prevent the onset, of schizophrenia.

Poly(I:C), a synthetic double-stranded RNA analog that mimics viral infection, activates innate immune signaling including TLR3-mediated pathways in microglia and other cell types. Poly(I:C)-induced maternal immune activation (MIA) is one of the most widely used and well-established in vivo models of schizophrenia. Its validity is supported by epidemiological evidence linking prenatal maternal infection and immune activation to increased schizophrenia risk, by the neurodevelopmental hypothesis of the disorder, and by its ability to reproduce schizophrenia-relevant behavioral and neuroimmune abnormalities [50]. Growing evidence indicates the therapeutic effects of minocycline in Poly(I:C)-MIA models, partly through inhibition of microglial activation. In Poly(I:C)-exposed offspring, minocycline has been shown to attenuate microglial activation and improve schizophrenia-like behavioral abnormalities, such as deficits in prepulse inhibition, social interaction, and locomotor regulation [51]. In a rat MIA model, minocycline has also been shown to normalize hippocampal microglia-generated proinflammatory cytokines, restore impaired adult hippocampal neurogenesis, and ameliorate sensorimotor gating [52,52]. In addition, in a neurodevelopmental two-hit model combining prenatal Poly(I:C) exposure with peripubertal stress, minocycline prevented the later emergence of behavioral dysfunctions while suppressing hippocampal and prefrontal microglial activation and IL-1β expression [53]. To our knowledge, however, only one study has indicated reduced dendritic spine density and increased microglial synaptic pruning in a Poly(I:C)-MIA model by showing the colocalization of immunoreactivity for postsynaptic density protein 95 and the microglial marker Iba-1 [54]. Importantly, this study did not investigate the effects of minocycline. Taken together, these preclinical findings provide a biological rationale for examining whether the microglia-modulating effects of minocycline on neuroinflammation and synaptic pathology can be translated into clinical benefits in patients with schizophrenia.

Microglial abnormalities in schizophrenia need not represent a primary disease-driving mechanism. Altered microglial responses may arise secondary to neuronal or synaptic abnormalities, complement-related synaptic vulnerability, or prenatal immune disturbance, and may interact with rather than initiate disease processes. Therefore, the rationale for minocycline in schizophrenia is not that microglial activation alone explains the disorder, but that inflammatory or maladaptive microglial responses may contribute to neurodevelopmental and synaptic pathology in a subset of patients.

Early meta-analyses (on 4–8 RCTs) reported that adjunctive minocycline significantly improved overall symptoms, in particular, negative symptoms in schizophrenia, although with substantial heterogeneity [55,56,57]. Such positive findings were generally interpreted in light of the microglia-inhibitory and broader anti-inflammatory properties of minocycline. Subsequent broader meta-analyses (on 10–13 RCTs) still detected modest benefits, particularly for negative symptoms. However, these analyses also reported substantial heterogeneity and limited robustness [58,59]. Moreover, the inclusion of large negative RCTs (n = 200–207), such as BeneMin and the study by Weiser et al. (2019), has further weakened confidence in a consistent clinical benefit [60,61]. Taken together, the adjunctive therapeutic effect of minocycline in schizophrenia is likely to be modest and inconsistent rather than robustly reproducible, since the overall evidence remains too heterogeneous to support routine clinical use. In addition, evidence for minocycline monotherapy in schizophrenia appears to be absent.

## 6. Effects of Minocycline on Major Depressive Disorder

There are only a limited number of studies using in vitro or, more precisely, ex vivo microglial systems that appear to recapitulate aspects of MDD-related microglial pathology. In particular, several studies have examined microglia isolated from the brains of mice subjected to repeated or chronic social defeat stress. These ex vivo cultured microglia exhibited disease-relevant phenotypes, including heightened responsiveness to LPS with enhanced production of proinflammatory cytokines, increased phagocytic activity, and enriched expression of genes associated with inflammation, phagocytosis, oxidative stress, and extracellular matrix remodeling [62,63,64]. Nevertheless, these studies did not examine the effects of minocycline on these MDD-related microglial properties. Moreover, a substantial translational gap may exist between rodent stress models and human MDD, since a single-cell mass cytometry study showed that microglia isolated from postmortem brains of patients with MDD displayed a non-inflammatory phenotype with increased expression of homeostatic markers [65]. Therefore, direct in vitro evidence showing that minocycline modulates disease-relevant microglial phenotypes in MDD remains scarce.

Compared with the limited in vitro/ex vivo studies, a larger body of in vivo evidence indicates the antidepressant-like and microglia-inhibitory effects of minocycline in rodent MDD models. Adult rodent chronic stress paradigms, such as chronic mild/unpredictable mild stress and chronic social defeat stress, are well-established and widely used in MDD research, since they reproduce several stress-related behavioral and neurobiological features that are considered to have considerable relevance to the pathophysiology of MDD. In the paradigms, systemic minocycline treatment generally ameliorated depressive-like behaviors, including behavioral despair, anhedonia, social avoidance, and stress-induced cognitive impairment. These behavioral ameliorations were accompanied with attenuation of activated microglia whose signatures were shown by the upregulation of translocator protein, Iba-1, CD11b, CD68, NLRP3 inflammasome-signaling molecules, metabolites in the kynurenine pathway, and high mobility group box 1 [66,67,68,69,70]. Since this evidence is based on heterogeneous experimental designs, including differences in stress paradigms, dosing schedules, and behavioral endpoints, the preclinical literature most strongly supports minocycline as a candidate intervention for inflammation-linked or stress-sensitized depressive states rather than for MDD in general. Moreover, McKim et al. (2016) reported that minocycline suppressed microglial activation and monocyte recruitment after repeated social defeat stress, whereas the drug did not prevent social avoidance, despite improving memory impairment [71]. This finding cautions against the simple interpretation that “microglial inhibition equals antidepressant effect.”

Clinical evidence for minocycline in MDD has become less supportive over time. Importantly, minocycline has generally been investigated not as antidepressant monotherapy, but as an adjunctive or augmentation treatment. Early meta-analytic evidence based on three RCTs was encouraging and implied possible antidepressant effects of adjunctive minocycline [72]. However, subsequent meta-analyses focusing on treatment-resistant depression (TRD) reported in 2023 yielded mixed conclusions. One analysis reported a statistically significant reduction in overall depression severity (SMD, −0.59; 95% CI, −0.98 to −0.20), including a smaller effect in the TRD subgroup (SMD, −0.36; 95% CI, −0.64 to −0.09) [73]. By contrast, another meta-analysis focusing on depression not responding to first-line therapy found no significant difference in change in depression severity (mean difference, −3.93; 95% CI, −16.14 to 8.28), response rate, or remission rate [74]. A major turning point was the MinoTRD RCT by Hellmann-Regen et al. (2022), which represents the largest and most rigorous negative trial of adjunctive minocycline in TRD to date [75]. The MinoTRD trial is also important for evaluating the inflammatory-subtype hypothesis. Exploratory subgroup analyses in the trial, including stratification by baseline CRP, also failed to identify an inflammation-defined subgroup with clear minocycline benefit [75], although an earlier small study implied possible efficacy in patients with low-grade inflammation [76].

Consistent with this shift, the latest meta-analysis reached a negative conclusion, finding no significant superiority of minocycline over placebo for depression [77]. Accordingly, minocycline should not currently be considered as a general antidepressant for non-selected MDD or TRD, but rather as an investigational adjunctive immunomodulatory strategy. The inflammatory-depression hypothesis remains biologically attractive but clinically unconfirmed.

The preclinical and clinical evidence reviewed above is summarized in Table 1 and Table 2. Table 1 provides an overview of the experimental models, microglial or neuroinflammatory findings, and disease-relevant outcomes in preclinical studies. Table 2 summarizes the corresponding clinical evidence, including therapeutic outcomes and whether microglial activation was directly assessed in patients.

## 7. Conclusions

Minocycline has served as an important pharmacological tool for investigating the contribution of microglial activation and neuroinflammation to neuropsychiatric and neurodegenerative disorders. Across AD, LBD, schizophrenia, and MDD, preclinical studies have generally supported the ability of minocycline to inhibit microglial activation and to ameliorate disease-relevant behavioral, synaptic, or neuropathological abnormalities. These findings have provided a biological rationale for targeting microglia-mediated neuroinflammation as a therapeutic strategy across a broad spectrum of neuropsychiatric disorders.

Nevertheless, the clinical translation of positive preclinical findings has been inconsistent and often disappointing. In AD, minocycline failed to improve cognitive or functional outcomes despite robust preclinical evidence. In LBD and related parkinsonian disorders, clinical benefits have not been consistently demonstrated with tolerability issues observed in some trials. In schizophrenia, early clinical studies and meta-analyses suggested possible benefits, particularly for negative symptoms, but subsequent larger negative trials weakened confidence in a robust therapeutic effect. Similarly, in MDD, early encouraging clinical signals have been weakened by later meta-analytic evidence and by the negative MinoTRD trial. Although inhibition of microglial activation remains mechanistically attractive, current clinical evidence does not support minocycline as an established disease-modifying or symptom-improving treatment for these disorders.

A key unresolved issue is whether minocycline actually modulates disease-relevant microglial responses in the human brain. Direct assessment of microglial target engagement in patients remains difficult, because currently available imaging and fluid biomarkers provide indirect rather than definitive evidence of specific microglial responses. Although the MSA trial using ^11^C-PK11195 PET suggested reduced microglial activation in some minocycline-treated patients, this was not accompanied by clinical benefit [45]. Complementary approaches, including PET imaging and fluid biomarkers of neuroimmune activity, such as soluble triggering receptor expressed on myeloid cells 2, may provide additional information on neuroimmune changes, but these measures should not be interpreted as direct evidence that a specific disease-relevant microglial state has been suppressed. Thus, the limited clinical evidence for target engagement reflects both the design of previous trials and the inherent difficulty of assessing microglial responses in the living human brain.

Several general translational limitations should be considered across disorders. Preclinical studies often use relatively homogeneous experimental conditions and treatment schedules that begin before or near the onset of pathological changes, whereas clinical trials involve heterogeneous patients with established disease, variable illness duration, comorbidities, concomitant medications, and diverse inflammatory profiles. Species differences, age and sex, dosing regimen, treatment duration, CNS drug exposure, and possible publication bias may further contribute to the translational gap. These challenges are compounded by the context-dependent heterogeneity of microglial states across brain regions, disease stages, age, and species [12,13]. Importantly, these factors may differ across disorders. In AD and LBD-related neurodegenerative disorders, intervention generally occurs after substantial protein aggregation and neuronal injury are already established, whereas schizophrenia has a neurodevelopmental component involving earlier synaptic and immune-related processes. In MDD, patient heterogeneity and the restriction of inflammatory mechanisms to a subset of patients may be particularly important. Accordingly, “microglial activation” should not be regarded as a uniform therapeutic target across diseases; the clinical value of minocycline is likely to depend on whether the relevant microglial response is pathogenic, reversible, and present at the time of intervention.

The rationale for testing minocycline in schizophrenia and MDD arose from preclinical and clinical evidence implicating immune-inflammatory and microglia-related processes in subsets of these disorders. However, the inconsistent clinical findings reviewed here indicate that this rationale is unlikely to apply broadly across unselected patients.

Taken together, the available evidence does not support continued development of minocycline as a broadly applicable “microglial inhibitor” for unselected patients with neurodegenerative or psychiatric disorders. However, abandoning minocycline entirely may also be premature. Its future value may lie in mechanistically informed and biomarker-guided studies targeting patients or disease stages characterized by demonstrable inflammatory or maladaptive microglial responses, with direct assessment of target engagement whenever feasible.

## Figures and Tables

**Figure 1:**
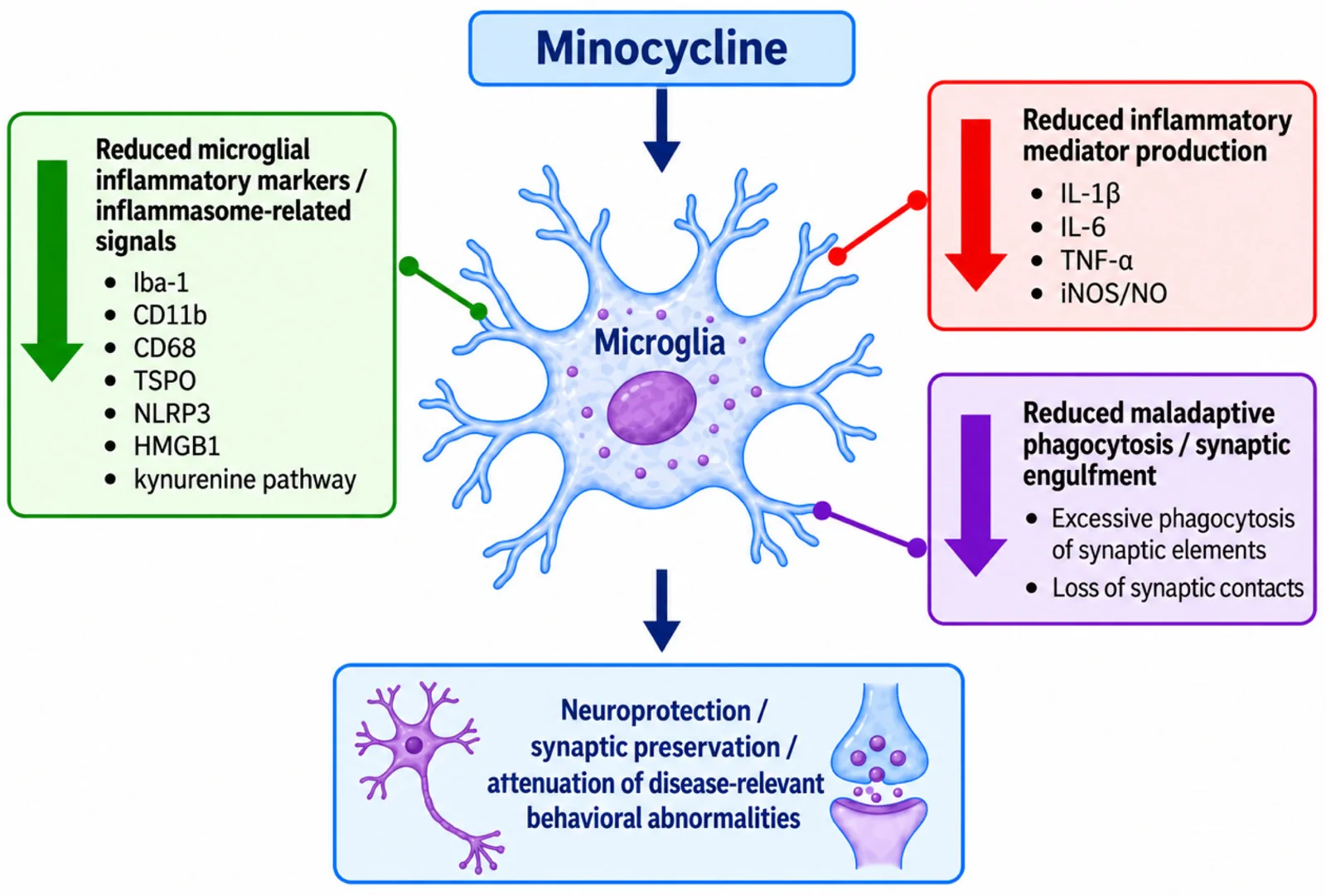
Putative mechanisms and downstream effects of minocycline on inflammatory or maladaptive microglial responses. Minocycline has been reported to reduce multiple readouts associated with microglial activation in preclinical models, including the production of inflammatory mediators, expression of microglial inflammatory markers and inflammation-related signals, and maladaptive phagocytosis or synaptic engulfment. These effects may contribute to neuroprotection, synaptic preservation, and attenuation of disease-relevant behavioral abnormalities. Importantly, these actions are not necessarily microglia-specific, because minocycline may also affect neurons, astrocytes, endothelial cells, and peripheral immune cells. Direct clinical evidence demonstrating target engagement by minocycline in the human brain remains limited. IL, interleukin; TNF-α, tumor necrosis factor-α; iNOS, inducible nitric oxide synthase; NO, nitric oxide; TSPO, translocator protein; NLRP3, NLR family pyrin domain containing 3; HMGB1, high mobility group box 1.

**Table 1:** Summary of preclinical evidence for minocycline in neuroinflammation-related neuropsychiatric disorders.

Disorder	Main Experimental Systems/Models	Microglial or Neuroinflammatory Findings	Disease-Relevant Outcomes	Main Interpretation/Limitations	References
Alzheimer’s disease	Aβ-stimulated human and mouse microglia; neuronal cultures exposed to Aβ; APP-transgenic, APP/PS1 double-transgenic, and triple-transgenic mice; Aβ-injected rat models	Minocycline reduced Aβ-induced IL-6, IL-1β, and TNF-α production from microglia without preventing Aβ phagocytosis. In animal models, it generally attenuated microgliosis.	Improved cognition, spatial learning and memory, and synaptic deficits in several models. Direct neuroprotective, anti-amyloidogenic, and anti-tauogenic effects were also reported in vitro.	Preclinical data support a protective role against microglial activation and neurotoxicity. However, effects on Aβ plaque burden and tau hyperphosphorylation were inconsistent, suggesting that benefit in animal models may depend more on glial/synaptic pathology and disease stage than on amyloid or tau burden alone.	[14,15,16,17,18,19,20,21,22,23,24,25,26,27]
Lewy body disease/Parkinsonian models	MPP+-stimulated BV2 cells and mixed neuron-glia cultures; MPTP, 6-OHDA, and intranigral LPS models; α-synuclein-transgenic mice	Minocycline inhibited MPP+-induced microglial activation, reduced caspase-1 and iNOS expression, and suppressed inflammatory mediators such as IL-1β, TNF-α, iNOS, NADPH oxidase, OX-42, and OX-6 in animal models.	Reduced dopaminergic neurodegeneration and preserved tyrosine hydroxylase-positive neurons in several models; improved motor function and attenuated Lewy body pathology in an α-synuclein-transgenic model.	Most studies support protective effects against microglial activation and dopaminergic neurodegeneration. However, a few studies reported neutral or deleterious effects, including worsened outcomes in MPTP-treated mice and monkeys, indicating context-dependent effects.	[30,31,32,33,34,35,36,37,38,39,40,41,42]
Schizophrenia	Patient-derived induced microglia-like cells and iPSC neuron/synaptosome systems; neuron–microglia co-cultures; Poly(I:C)-induced maternal immune activation models; neurodevelopmental two-hit models	Minocycline reduced microglial engulfment of synaptic structures in patient-derived cellular models and attenuated microglial activation and proinflammatory cytokine production in MIA models.	Rescued presynaptic density in schizophrenia-derived co-cultures; improved schizophrenia-like behavioral abnormalities, including deficits in prepulse inhibition, social interaction, locomotor regulation, and sensorimotor gating; restored impaired adult hippocampal neurogenesis in some models.	Preclinical findings provide a rationale for targeting microglia-mediated synaptic pathology and neuroinflammation. However, direct evidence that minocycline suppresses excessive microglial synaptic pruning in in vivo schizophrenia models remains limited.	[46,46,47,48,49,50,51,52,52,53,54]
Major depressive disorder	Ex vivo microglia isolated from repeated/chronic social defeat stress models; chronic mild/unpredictable mild stress and chronic social defeat stress models	Stress-exposed microglia showed heightened LPS responsiveness, increased phagocytic activity, and enriched expression of genes related to inflammation, phagocytosis, oxidative stress, and extracellular matrix remodeling. In vivo minocycline generally attenuated activated microglial signatures, including TSPO, Iba-1, CD11b, CD68, NLRP3 inflammasome signaling, kynurenine pathway dysregulation, and HMGB1-related pathways.	Ameliorated depressive-like behaviors, including behavioral despair, anhedonia, social avoidance, and stress-induced cognitive impairment in several rodent models.	Preclinical evidence supports minocycline as a candidate intervention for inflammation-linked or stress-sensitized depressive states rather than for MDD in general. McKim et al. reported suppression of microglial activation and monocyte recruitment without prevention of social avoidance, cautioning against a simple ‘microglial inhibition equals antidepressant effect’ interpretation.	[62,63,64,66,67,68,69,70,71]

Abbreviations: Aβ, amyloid β; APP, amyloid precursor protein; HMGB1, high mobility group box 1; IL, interleukin; iNOS, inducible nitric oxide synthase; iPSC, induced pluripotent stem cell; LPS, lipopolysaccharide; MDD, major depressive disorder; MIA, maternal immune activation; MPP+, 1-methyl-4-phenylpyridinium; MPTP, 1-methyl-4-phenyl-1,2,3,6-tetrahydropyridine; NLRP3, nucleotide-binding oligomerization domain-like receptor pyrin domain-containing 3; PS1, presenilin-1; TNF, tumor necrosis factor; TSPO, translocator protein; 6-OHDA, 6-hydroxydopamine.

**Table 2:** Summary of clinical evidence for minocycline in neuroinflammation-related neuropsychiatric disorders.

Disorder/Clinical Setting	Study Type and Population	Minocycline Regimen/Study Design	Main Clinical Findings	Direct Assessment of Microglial Activation in Patients	Main Interpretation	References
Alzheimer’s disease	Randomized controlled trial in patients with mild AD	Daily minocycline 200–400 mg for 2 years vs. placebo	No meaningful benefit in cognition, assessed by Mini-Mental State Examination, or functional ability, assessed by Bristol Activities of Daily Living Scale.	Not assessed	Despite robust preclinical evidence, minocycline failed to show clinical efficacy in mild AD. No direct evidence was provided that minocycline suppressed microglial activation in the human AD brain.	[29]
Parkinson’s disease/early parkinsonian disease	NINDS NET-PD phase II futility trial and 18-month follow-up	Creatine or minocycline evaluated in early PD	Initial futility trial suggested possible benefit, but follow-up showed no significant differences from placebo. Tolerability concerns led to discontinuation in 23% of participants.	Not directly assessed	Clinical benefit was not consistently demonstrated, and tolerability was a concern.	[43,44]
Multiple-system atrophy, parkinsonian type	MEMSA trial in patients with MSA	One-year minocycline therapy	Failed to improve motor function; high dropout rate due to adverse effects, including gastrointestinal intolerance, worsening parkinsonism, and skin hyperpigmentation.	11C-(R)-PK11195 PET suggested reduced microglial activation in some minocycline-treated patients.	This is a rare clinical example in which microglial activation was directly assessed by PET, but the imaging finding did not translate into clinical benefit.	[45]
Schizophrenia	Early and subsequent meta-analyses of adjunctive minocycline RCTs; later large negative RCTs	Adjunctive minocycline added to antipsychotic treatment	Early meta-analyses suggested improvement in overall symptoms, especially negative symptoms. Subsequent broader meta-analyses suggested modest effects but with substantial heterogeneity. Large negative RCTs, including BeneMin and the study by Weiser et al., weakened confidence in robust efficacy.	Not directly assessed in clinical trials	Adjunctive minocycline may have modest and inconsistent effects, especially on negative symptoms, but evidence is too heterogeneous to support routine clinical use. Evidence for minocycline monotherapy appears absent.	[55,56,57,58,59,60,61]
Major depressive disorder/treatment-resistant depression	Systematic reviews, meta-analyses, and RCTs of minocycline in depression and TRD	Mostly adjunctive or augmentation treatment added to ongoing antidepressant therapy or treatment as usual	Early meta-analysis based on three RCTs was encouraging. Later meta-analyses yielded mixed conclusions. The MinoTRD trial, the largest negative RCT of adjunctive minocycline in TRD, found no superiority over placebo. The latest meta-analysis found no significant superiority over placebo for depression.	Not directly assessed in most trials; CRP-based subgroup analyses in MinoTRD failed to identify an inflammation-defined subgroup with clear benefit.	Current evidence does not support minocycline as a general antidepressant for unselected MDD or TRD. The inflammatory-depression hypothesis remains biologically attractive but clinically unconfirmed.	[72,73,74,75,76,77]

Abbreviations: AD, Alzheimer’s disease; CRP, C-reactive protein; MDD, major depressive disorder; MEMSA, Minocycline in Multiple-System Atrophy trial; MSA, multiple-system atrophy; PD, Parkinson’s disease; PET, positron emission tomography; RCT, randomized controlled trial; TRD, treatment-resistant depression.

## Data Availability

No new data were created or analyzed in this study. Data sharing is not applicable to this article.
